# Transcriptional-profile changes in the medial geniculate body after noise-induced tinnitus

**DOI:** 10.3389/ebm.2024.10057

**Published:** 2024-03-18

**Authors:** Peng Liu, Xinmiao Xue, Chi Zhang, Hanwen Zhou, Zhiwei Ding, Li Wang, Yuke Jiang, Wei-Dong Shen, Shiming Yang, Fangyuan Wang

**Affiliations:** ^1^ Medical School of Chinese PLA, Beijing, China; ^2^ Department of Otolaryngology, Head and Neck Surgery, Institute of Otolaryngology, Chinese PLA General Hospital, Beijing, China; ^3^ National Clinical Research Center for Otolaryngologic Diseases, Beijing, China

**Keywords:** tinnitus, transcriptional profile, RNA-sequencing, medial geniculate body, gene

## Abstract

Tinnitus is a disturbing condition defined as the occurrence of acoustic hallucinations with no actual sound. Although the mechanisms underlying tinnitus have been explored extensively, the pathophysiology of the disease is not completely understood. Moreover, genes and potential treatment targets related to auditory hallucinations remain unknown. In this study, we examined transcriptional-profile changes in the medial geniculate body after noise-induced tinnitus in rats by performing RNA sequencing and validated differentially expressed genes via quantitative polymerase chain reaction analysis. The rat model of tinnitus was established by analyzing startle behavior based on gap-pre-pulse inhibition of acoustic startles. We identified 87 differently expressed genes, of which 40 were upregulated and 47 were downregulated. Pathway-enrichment analysis revealed that the differentially enriched genes in the tinnitus group were associated with pathway terms, such as coronavirus disease COVID-19, neuroactive ligand-receptor interaction. Protein–protein-interaction networks were established, and two hub genes (Rpl7a and AC136661.1) were identified among the selected genes. Further studies focusing on targeting and modulating these genes are required for developing potential treatments for noise-induced tinnitus in patients.

## Introduction

Tinnitus, a condition in which people with or without hearing loss perceive phantom sounds, has become a major problem affecting millions of people worldwide. Research related to the epidemiology of tinnitus has demonstrated that nearly 25% of all Americans experience abnormal auditory sensations at least once in their lifetime [1]. When tinnitus becomes chronic (>6 months), various co-morbidities including insomnia, and psychological disorders, such as anxiety, depression, cognitive dysfunction, and stress, influence the quality of patients’ lives and even lead to suicide [2]. Although frequently caused by hearing loss and aging, tinnitus is hearing loss- and age-independent, suggesting that an extremely intricate mechanism mediates the onset of this disease [3]. Current therapeutic strategies for treating tinnitus (including drugs, acoustic stimulation, psychological therapy, and repetitive transcranial magnetic stimulation) have generated conflicting evidence regarding beneficial outcomes and alleviation of the disease [4].

Since tinnitus can persist after the destruction of the auditory nerve, recent studies have attributed the generation of tinnitus to the central auditory pathway, which involves the auditory cortex, medial geniculate body (MGB), inferior colliculus, and cochlear nucleus, instead of the peripheral otologic components [5]. The MGB, an obligate auditory brain center, plays an essential role in transmitting acoustic information from the inferior colliculus to the auditory cortex [6]. Not only is the MGB the principle conduit between the thalamic circuits and the cortex, but that there is evidence for altered firing patterns along this ascending input in animal models of tinnitus [7]. Based on its anatomical features, the MGB is a suitable candidate region for studies related to tinnitus [8]. Previous research indicated that the firing of neurons in the MGB changes from the tonic to burst form, which was found to be a significant indicator of hyperactivity after tinnitus [9]. Inhibition of the abnormal response can equally alleviate oscillations induced by tinnitus between the MGB and auditory cortex [10]. Altered biological function of the MGB can lead to abnormal signal transmissions in the auditory pathways, which can subsequently mediate the development of tinnitus.

In the context of biological traits, genes are important hereditary units that modulate numerous life processes, including birth, illness, and death. Recent advancements in genomics have provided insights into relationships between genes associated with different human diseases and genetic heredity in tinnitus [11]. Animal transcriptomics studies have revealed several genes that are expressed differently after tinnitus, such as NR2B, VGLUT1, BDNF, and Gabrb3 [12–15]. In addition, the results of some studies on patients with tinnitus showed that the expression levels of genes related to cardiovascular function, neurotrophic factors, GABAB receptor subunits, and serotonin transporter function were significantly increased [16–19]. Meanwhile, human genetic studies on tinnitus that have been replicated in an independent cohort also revealed that genes, such as *AF131215.5, BLK, C8orf12, COL11A1, GRK6, MSRA, MFHAS1, XKR6, ANK2, AKAP9,* and *TSC2*, could act as major predictors of the development of tinnitus [11, 20, 21]. Although abundant data have been generated regarding the genetic underpinnings of tinnitus, differentially expressed genes (DEGs) in the MGB remain unclear for this clinical enigma, which limits the development of effective treatments. Exploring potential genes in brain regions that underlie tinnitus vulnerability would lay a foundation for understanding tinnitus pathogenesis and exploring effective intervention strategies.

In this study, we performed RNA-sequencing (RNA-seq) to identify DEGs in the MGB that correlated with noise-induced tinnitus and elucidated related signaling pathways. Two hub genes (*Rpl7a* and *AC136661.1*) in the MGB showed potential as effective therapeutic drug targets.

## Materials and methods

### Animals

Two-month-old male Sprague–Dawley rats were housed in standard cages (12 h day/night cycle) with a normal humidity (50–60%) and an appropriate temperature (22°C) with food and water *ad libitum*. All procedures were performed in accordance with the requirements of the Care and Use of Laboratory Animals of the Chinese PLA General Hospital. All protocols used in this study were approved by the Animal Ethics Committee of the Chinese PLA General Hospital (Code: 2021-X17-85).

### Auditory brainstem responses (ABRs)

The animals included in this study were verified by performing ABR tests, which can be used to study noise exposure. Briefly, the rats were administered sodium pentobarbital intraperitoneally (i.p.) and subsequently placed in a soundproof chamber. Tone and click stimuli (0.5 ms rise or fall) were applied using a TDT loudspeaker (Tucker Davis Technologies, Miami, FL, United States). A tube linked to a TDT RZ6 instrument was placed in the external auditory canal. Before ABR testing, the animals were administered reference, active, and ground needle electrodes (Rochester Elektro-Medical, Lutz, FL, United States). The reference needle was placed on the tested mastoid, whereas the ground needle was set contralaterally, and the active needle inserted into the vertex of the skin. The original sound-pressure level (SPL) was set at 90 dB and then it was decreased in 10 dB steps for both the click and tone stimuli (4, 8, 16, and 32 kHz). The recorded amplified responses were filtered through a passband from 100 Hz to 3 kHz and averaged 512 times. To detect the ABR threshold, repeatable wave-III patterns were monitored at every frequency until they disappeared with decreasing SPLs, and the lowest dB SPL at each frequency was recorded.

### Gap detection

The establishment of tinnitus in animals after noise exposure was verified by assessing gap-induced pre-pulse inhibition (PPI) of the acoustic startle responses, as described previously [22]. Briefly, a cage connected to a piezoelectric transducer was used to detect pressure changes caused by acoustic startles and instantaneously transform them into voltage values that were utilized to evaluate the amplitudes of the acoustic startle response (ASR). The “no-gap” pattern was set using background noise (60 dB SPL) centered at 6, 12, or 16 kHz, inserted with a 115 dB SPL startle stimulus lasting for 20 ms. The “gap” pattern was set using a silent cap (50 ms) delivered 100 ms ahead of the startle stimulus onset, and the results were compared with those obtained using the no-gap pattern. A speaker (controlled using startle software, Xeye, Beijing, China) was installed 20 cm above the platform and linked to the piezoelectric transducer to generate auditory stimuli. Ten paired “gap” and “no-gap” trials were conducted in random order for all tests. The ability of animals to detect gap was estimated by gap: PPI (%) ratio, which was calculated as amplitude of the 1-gap ASR divided by the amplitude of the no-gap ASR. The criteria for tinnitus was as follows: At least the startle ratio of single test frequency before exposure was more than 30% before noise exposure and the startle ratio after exposure is required to be below 30%. In addition, decrease in startle ratio for a single frequency should be more than 30% [23]. Otherwise, the animals are considered as non-tinnitus ones.

### Noise exposure

Rats wearing foam earplugs (OHRFRIEDEN, Wehrheim, Germany) unilaterally in the right ear were used in this study. The rats were exposed to loud noise at a frequency from 8–16 kHz (126 dB SPL) for 2 h after being deeply anesthetized with an i.p. injection of sodium pentobarbital [24]. Unilateral noise exposure allowed animals to maintain normal hearing, which was essential for gap detection. The sound-delivery system comprised an RA 300 amplifier (Alesis, Cumberland, RI, United States) and a TW67 speaker (Pyramid Car Audio, Brooklyn, NY, United States). Briefly, the speaker was arranged 10 cm away from the ears of each rat, and the RA 300 amplifier and TDT processor were arranged to generate and amplify the tones. Calibration of the SPL was achieved using a sound-level meter connected to a condenser microphone.

### RNA-seq analysis

RNA-seq was performed as described elsewhere [25]. Regions (5.2–6.36 mm posterior to bregma; 3.2–4.2 mm, lateral to the midline; 5.2–6.8 mm ventral to the dorsal surface of the skull) were selected as the target area according to brain atlas of rats. Briefly, MGB tissue samples (*n* = 3) acquired from the non-tinnitus and tinnitus groups were washed immediately, and processed for RNA isolation using TRIzol reagent (Thermo Fisher Scientific, Wilmington, DE, United States). The RNA concentration and purity of each sample were measured using a NanoDrop 2000 spectrophotometer (Thermo Fisher Scientific). The RNA Nano 6000 Assay Kit was used to assess RNA integrity. Sequencing libraries were generated using the NEBNext UltraTM RNA Library Prep Kit for Illumina (New England Biolabs, United States) according to the manufacturer’s recommendations, and index codes were added to attribute the sequences to each sample. Raw data (raw reads) in fastq format were first processed using in-house Perl scripts. To obtain clean data (clean reads), reads containing poly-N sequences, adapters, and low-quality reads were removed from the raw data. Then, the Q20, Q30, GC content, and sequence-duplication levels of the clean data were calculated. After cleaning the data, high-quality data were acquired and downstream analyses were performed.

### Bioinformatics analysis

Edge R and DEseq2 packages were used to analyze differential gene expression in tissue samples. A *p*-value of <0.05 and a fold-change of ≥1.5 were used as criteria for identifying genes that are significantly modulated levels [26]. The GOseq R package and KOBAS software were used to analyze enriched processes and pathways identified using the Gene Ontology (GO) and Kyoto Encyclopedia of Genes and Genomes (KEGG) databases, respectively. The DEGs were uploaded to the STRING database,^1^ and protein–protein-interaction analysis was performed. Cytoscape software was used to visualize and select hub genes.

### Quantitative polymerase chain reaction (qPCR) analysis

All experimental procedures for RNA extraction used in this study were in accordance with those employed in previous studies [27]. Total RNA (*n* = 5) was extracted using RNA Extraction Reagent (Servicebio, Wuhan, China) and converted to complementary DNA (cDNA) using the Servicebio RT First Strand cDNA Synthesis Kit (Servicebio). qPCR analysis was performed using 2× SYBR Green qPCR Master Mix (Servicebio). The mRNA-expression levels of the target genes were normalized to glyceraldehyde-3-phosphate dehydrogenase (GAPDH) mRNA-expression levels and fold-changes in expression differences were calculated using the 2^−ΔΔCT^ method [28]. The sequences of the oligonucleotide primers (Servicebio) used in this study are shown in Table 1.

**TABLE 1:** Sequences of primers used for qPCR analysis.

Gene	Forward primer (5′–3′)	Reverse primer (5′–3′)
*Fau*	GAC​GGT​CGC​CCA​GAT​CAA​A	GGT​TGT​ACT​GCA​TTC​GCC​TCT​T
*Rpl7a*	GAC​AAG​GGT​GCT​CTG​GCT​AAG	GCA​ATG​CGA​GCC​ACA​GAC​TTA
*Rps19*	AAC​CAG​CAG​GAG​TTC​GTC​AGA	ACC​ACC​ACG​GAG​GTA​CAG​GT
*LOC100360491*	TTC​TCC​TCT​TCC​GTG​ATG​GCT	ATC​CAC​AAG​AAA​ATG​GCA​CGC
*LOC685085*	AAA​GAA​GAA​GTG​GTC​CAA​AGG​CA	CTG​TGC​TTT​GAA​ACC​AGC​TTG​AT
*AC136661.1*	TAC​CTG​TTC​TCC​CTG​CCC​ATT	GTA​GTC​CCC​AAT​AGC​GAC​AAA
*Fos*	TCC​AAG​CGG​AGA​CAG​ATC​AAC​T	TCA​AGT​CCA​GGG​AGG​TCA​CAG​A
*Ebna1bp2*	AAG​AAG​GCG​GTG​AAT​GAC​GA	GCA​AAA​TAA​TCA​GTG​GGC​CTC​TT
*Egr1*	CCA​AAC​TGG​AGG​AGA​TGA​TGC​T	GAC​TCT​GTG​GTC​AGG​TGC​TCG​TA
*LOC689899*	AAC​AAG​CAC​CAG​ATC​AAA​CAG​G	TGG​CAA​CAT​CTA​GAG​CAT​CAT​AAT​C
*Gapdh*	CTG​GAG​AAA​CCT​GCC​AAG​TAT​G	GGT​GGA​AGA​ATG​GGA​GTT​GCT

### Statistical analysis

GraphPad Prism software (version 9.0.1; San Diego, CA, United States) was used to analyze the behavioral data generated in this study. The ABR thresholds and changes in GAP-PPI ASR were assessed using two-way ANOVA. Changes in the mRNA level determined using qPCR were examined using non-paired Student’s t-test. Data are presented as the mean ± standard error of the mean. *p* < 0.05 was applied as the threshold for statistical significance.

## Results

### Validation of tinnitus established by noise in rats

**FIGURE 1:**
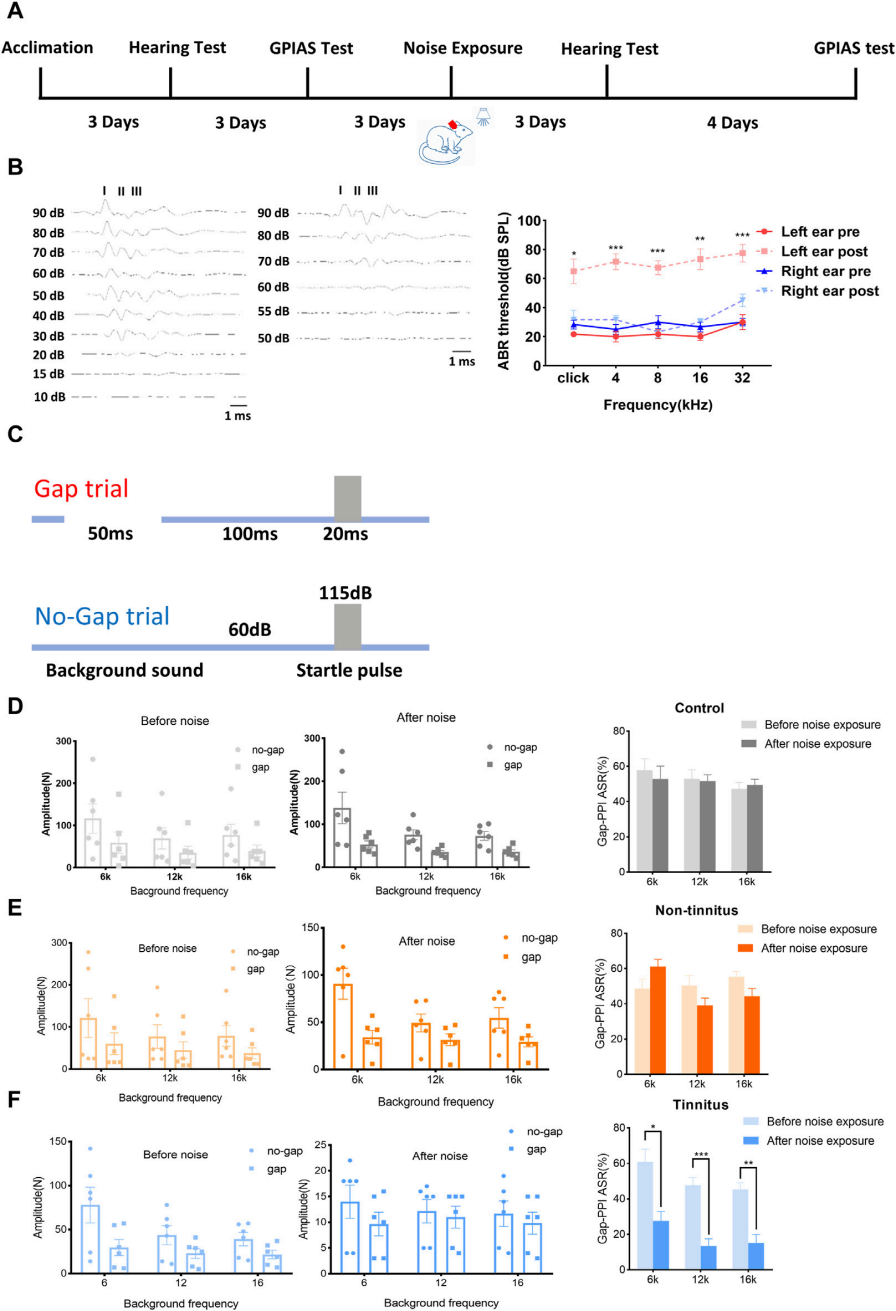
Establishment of a rat model of tinnitus induced by loud noise. **(A)** Schematic illustration depicting the experimental timeline. **(B)** Typical image of the ABR threshold upon click stimulation of left ear before (left) and after noise (middle) exposure. The ABR threshold of the left ear after noise exposure was significantly increased after click stimulation or exposure to a tone with a frequency of 4, 8, 16, or 32 kHz (right). **(C)** The experimental paradigm for detecting tinnitus. **(D–F)** Gap-PPI values of **(D)** the control, **(E)** non-tinnitus and **(F)** tinnitus groups at 6, 12, or 16 kHz. *n* = 6 animals. *p < 0.05, **p < 0.01, ***p < 0.001.

Figure 1A shows the experimental design of our study. Figure 1B (left panel) shows that the acoustic threshold of the left ear following a significant increase in the noise level. As depicted in Figure 1B (right panel), the acoustic threshold of the left ear (following the noise increase [post]) was significantly higher than before (pre) the noise when the click stimulus was introduced (pre vs. post, 21.67 ± 1.67 vs. 65.00 ± 8.47, *p* < 0.01). Similar findings were observed with different tone stimuli, including 4 kHz (pre vs. post, 20.00 ± 3.65 vs. 71.67 ± 5.43, *p* < 0.01), 8 kHz (pre vs. post, 21.67 ± 3.07 vs. 67.50 ± 4.79, *p* < 0.01), 16 kHz (pre vs. post, 20.00 ± 2.58 vs. 73.33 ± 7.15, *p* < 0.001), and 32 kHz (pre vs. post, 30.00 ± 5.16 vs. 77.5 ± 6.02, *p* < 0.01). The hearing threshold of the right ear was not significantly different after initiating click stimuli (pre vs. post, 28.33 ± 3.07 vs. 31.67 ± 6.54, *p* = 0.66) or tone stimuli, i.e., 4 kHz (pre vs. post, 25.00 ± 3.42 vs. 31.67 ± 3.07, *p* = 0.17), 8 kHz (pre vs. post, 30.00 ± 4.47 vs. 23.33 ± 4.22, *p* = 0.33), 16 kHz (pre vs. post, 26.67 ± 3.33 vs. 30.00 ± 2.58, *p* = 0.58), and 32 kHz (pre vs. post, 30.00 ± 2.58 vs. 45.33 ± 4.28, *p* = 0.06). Unilateral normal hearing enables animals to detect gaps in background sounds. The experimental design used to assess the ability of the animals to detect gaps is shown in Figure 1C. Animals in the control group exhibited comparable Gap-PPI changes at 6 kHz (pre vs. post, 57.82 ± 6.55 vs. 52.79 ± 7.41%, *p* = 0.63), 12 kHz (pre vs. post, 52.96 ± 5.15 vs. 51.74 ± 3.53%, *p* = 0.88), and 16 kHz (pre vs. post, 47.31 ± 3.56 vs. 49.47 ± 3.24%, *p* = 0.68), as shown in Figure 1D. These findings were equally applicable to the non-tinnitus group, which displayed no significant change at 6 kHz (pre vs. post, 48.74 ± 5.29 vs. 61.24 ± 4.08, *p* = 0.11), 12 kHz (pre vs. post, 50.53 ± 5.58 vs. 39.11 ± 4.18, *p* = 0.21), and 16 kHz (pre vs. post, 55.50 ± 2.89 vs. 44.30 ± 4.47, *p* = 0.12) in terms of gap-PPI detection (Figure 1E). In the tinnitus group, the inhibitory effect of the gap on acoustic startling was attenuated at 6 kHz (pre vs. post, 60.99 ± 7.07 vs. 27.63 ± 5.31, *p* < 0.01, Figure 1F). At 12 kHz, the tendency of decreasing inhibition was also observed, where the gap-PPI decreased from 47.67 ± 4.45% to 13.57 ± 4.03% (*p* < 0.01, Figure 1F). This type of change was equally applicable to a 16 kHz background sound, where the gap-PPI of the tinnitus group decreased from 45.38 ± 3.69% to 15.20 ± 4.79% (*p* < 0.01, Figure 1F). In order to exclude the potential loss of hearing in the plugged ear via damage to binaural ascending afferents [29], the ABR threshold of non-tinnitus and tinnitus group was tested which were shown in Supplementary Figures S1A, S1B. There was a similar ABR threshold change in the non-tinnitus and tinnitus group.

### Identification of DEGs

**FIGURE 2:**
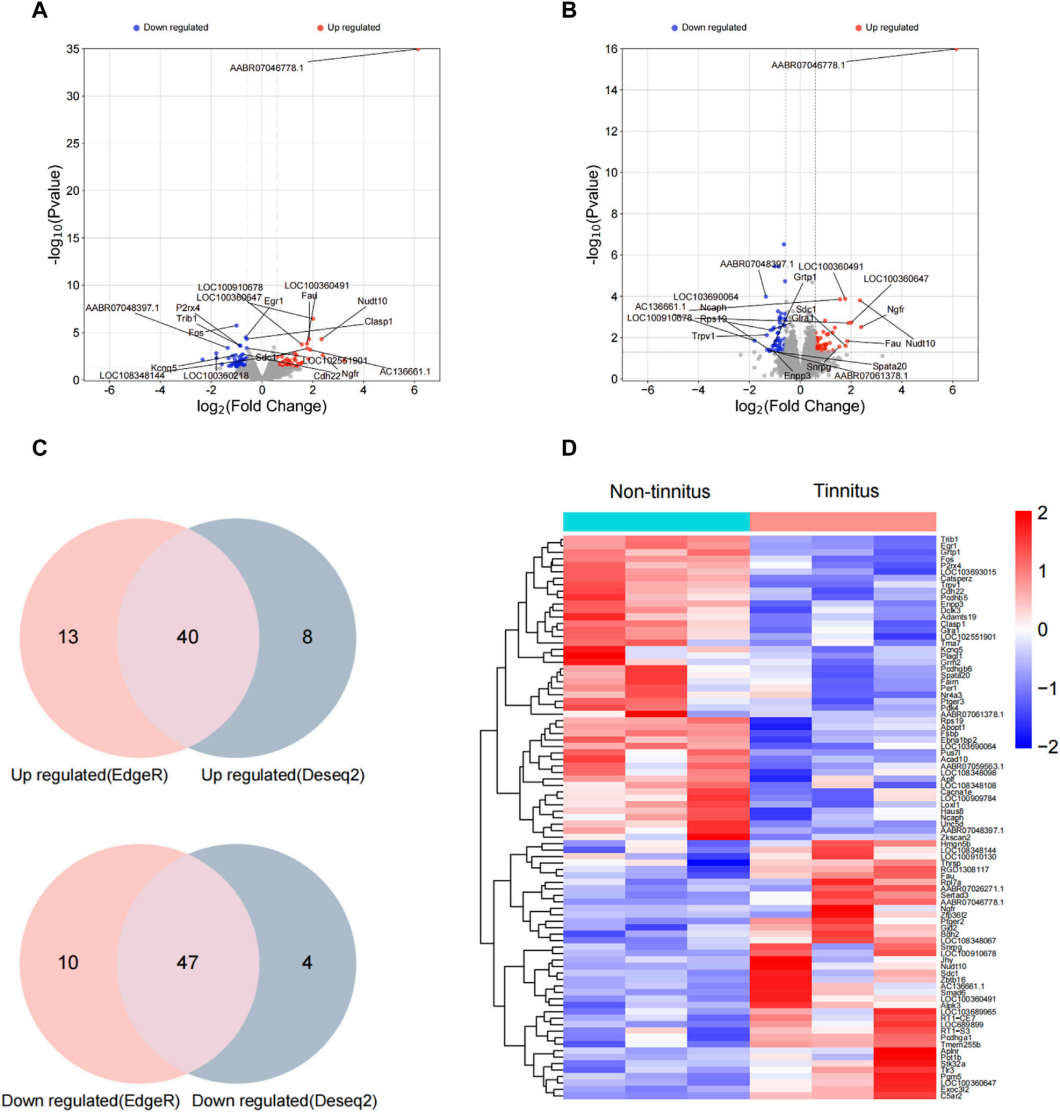
Confirmation of differential gene expression between the non-tinnitus and tinnitus groups at the transcriptional level. **(A,B)** Differently expressed genes as screened out by Edger and Deseq2. **(C)** Venn plots of differently expressed genes screened by Edge R and DESeq2. **(D)** Heatmap showing DEGs in the non-tinnitus (left) and tinnitus (right) groups. Blue and red colors indicate downregulated and upregulated DEGs, respectively. *n* = 3 animals.

To illuminate transcriptional-profile changes associated with noise-induced tinnitus, we performed RNA-seq analysis of brain samples from the non-tinnitus and tinnitus groups. The results of numerous studies related to tinnitus have shown that the MGB plays an important role in the development of this disease [30]; therefore, the MGB was chosen as the target area. Genes exhibited expression differences of >1.5-fold between the non-tinnitus and tinnitus groups were designated as potential DEGs. 110 DEGs and 99 DEGs were identified by EdgeR and DEseq2, respectively (Figures 2A, B). DEGs selected by EdgeR contained 53 upregulated and 57 downregulated. DEGs selected by DEseq2 contained 48 upregulated and 51 downregulated. By integrating the results from EdgeR and DEseq2, we identified 87 differently expressed genes which include 40 upregulated DEGs and 47 downregulated DEGs (Figure 2C). The selected DEGs are shown in a heat map (Figure 2D). Top ten upregulated and downregulated DEGs selected by Edger and Deseq2 were shown in Table 2.

**TABLE 2:** The top ten upregulated and downregulated DEGs.

Up regulated genes	log2FoldChange	False-discovery rate	*p*-value	Down regulated genes	log2FoldChange	False-discovery rate	*p*-value
*AABR07046778.1*	6.136147593	1.72E-31	1.03E-16	*LOC103690064*	−1.802058571	1	0.014442155
*Ngfr*	2.387695704	0.967933878	0.003147254	*AABR07048397.1*	−1.353588127	0.299396874	0.000105157
*Nudt10*	2.345152966	0.061686258	0.000159045	*Trpv1*	−1.321438256	1	0.007588696
*LOC100360647*	2.004777335	0.001272584	0.001899531	*AABR07061378.1*	−1.310732471	1	0.039494665
*AC136661.1*	1.900711856	0.37144022	0.001977346	*Ncaph*	−1.220065263	1	0.039471337
*Fau*	1.847325179	0.061686258	0.015040405	*Spata20*	−1.206301061	1	0.045839587
*Sdc1*	1.783174086	0.299396874	0.025586079	*Rps19*	−1.166814696	1	0.004378735
*LOC100360491*	1.764732106	0.146915516	0.000136002	*Enpp3*	−1.104953628	1	0.03958059
*LOC100910678*	1.562883998	0.164957053	0.000141141	*Grtp1*	−1.088605849	1	0.026009829
*Snrpg*	1.533385569	1	0.027685201	*Glra1*	−1.076531654	1	0.004025429

### DEG-enrichment analysis

**FIGURE 3:**
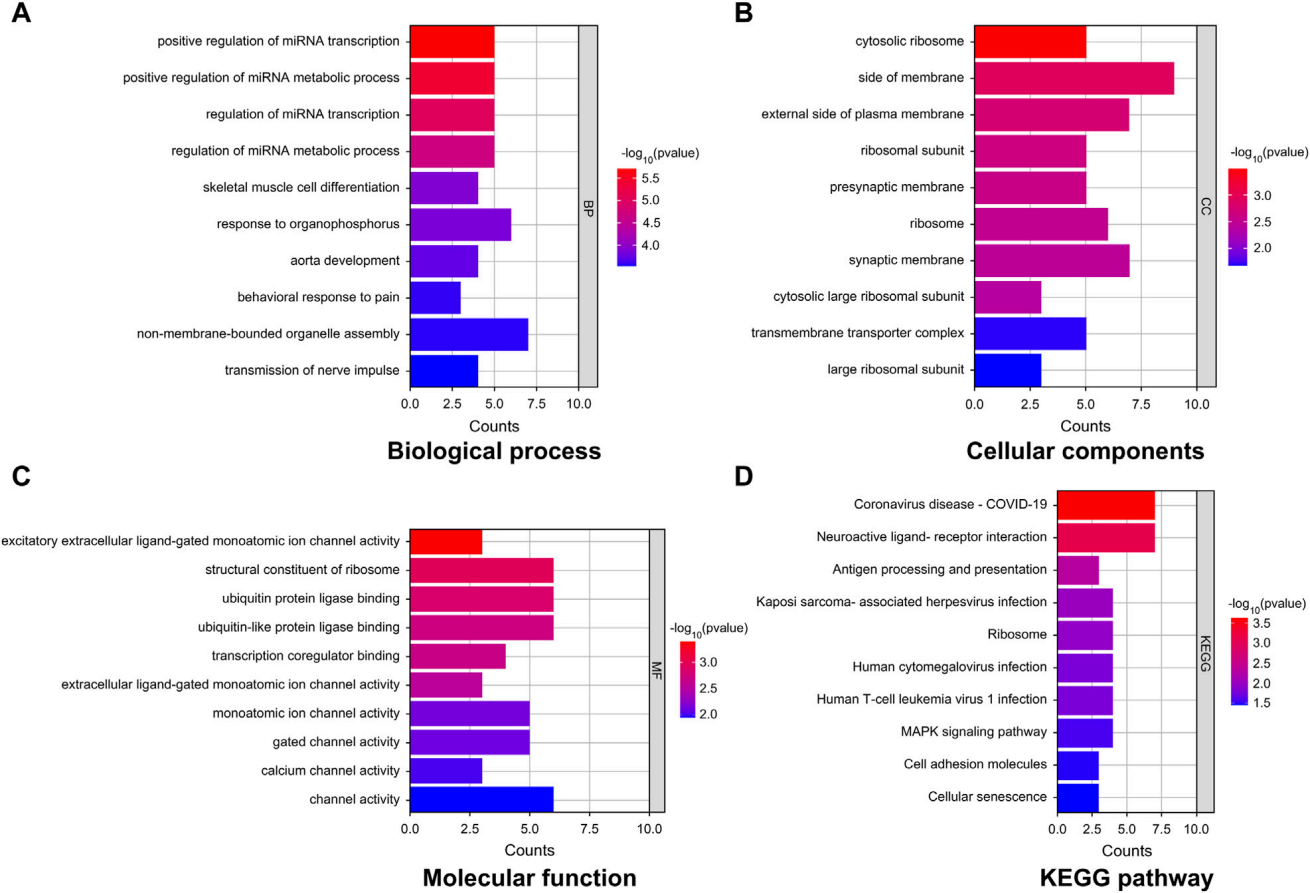
GO and KEGG enrichment analyses of key mRNAs. **(A–C)** Representative GO analysis of DEGs enriched for **(A)** Biological Process, **(B)** Cellular Components, and **(C)** Molecular Function. **(D)** The most highly enriched pathways associated with DEGs.

GO-based enrichment analysis was performed to assess the relevant biological functions of the DEGs in the non-tinnitus and tinnitus groups. Figures 3A–C depicts the enrichment terms in the biological process (BP), cellular component (CC), and molecular function (MF) categories. Three top most BP terms were positive regulation of miRNA transcription, positive regulation of miRNA metabolic process and regulation of miRNA transcription. The most enriched CC term was cytosolic ribosome, followed by side of membrane and external side of plasma membrane with the latter two terms were not significantly enriched. The top three most enriched MF terms were excitatory extracellular ligand-gated monoatomic ion channel activity, structural constituent of ribosome and ubiquitin protein ligase binding with the latter two terms showing no statistical significance. KEGG analysis was performed to elucidate the underlying pathways associated with the DEGs between the tinnitus and non-tinnitus groups. The most significantly enriched pathways identified were coronavirus disease COVID-19 and neuroactive ligand-receptor interaction (Figure 3D).

### Construction of a protein–protein-interaction network and selection of hub genes

**FIGURE 4:**
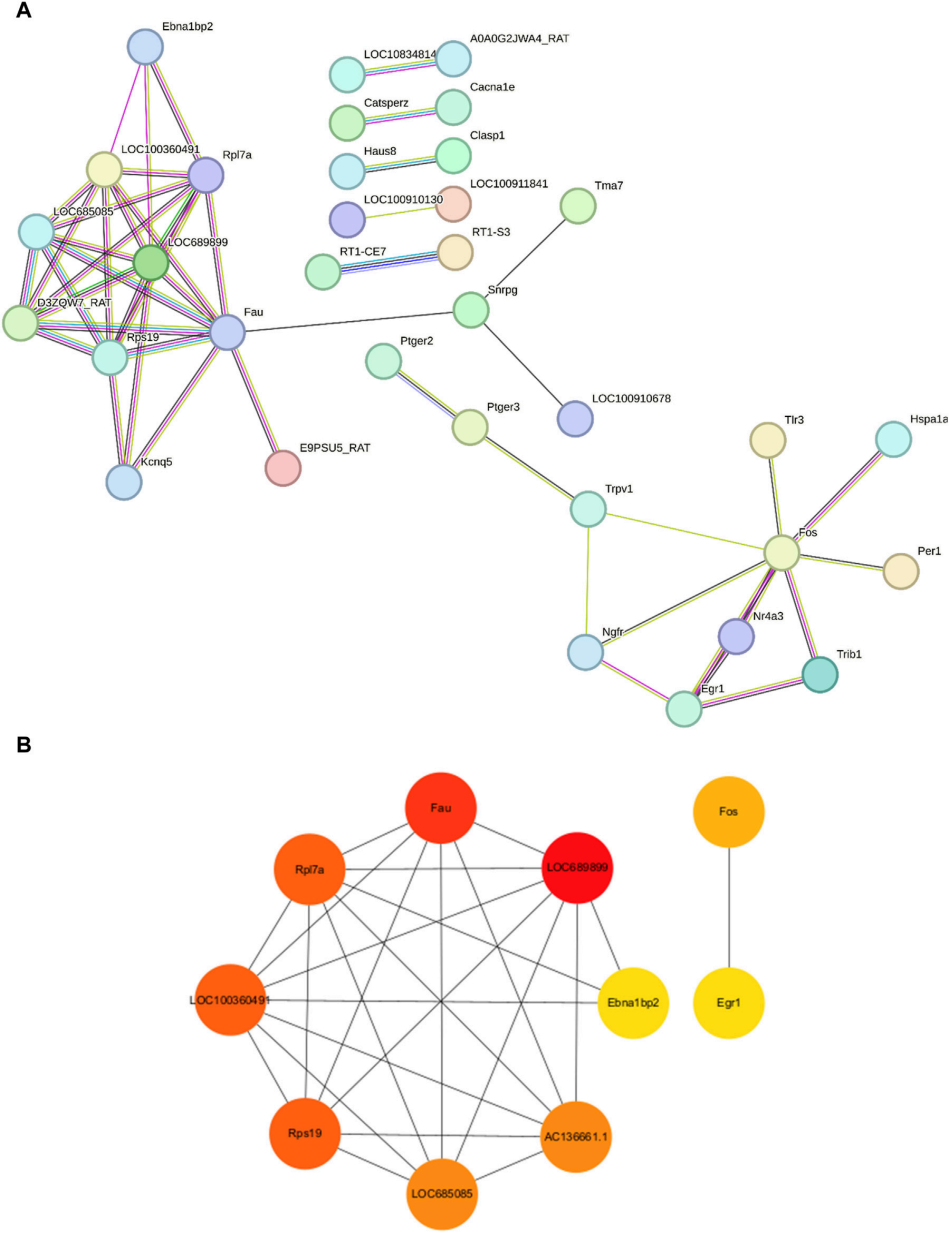
Protein–protein-interaction network in the experimental model of tinnitus **(A)** The protein–protein-interaction network established using the STRING database and the hub genes identified in this study. **(B)** Key genes are arranged based on their degree values. The top ten genes were selected as potential hub genes.

We employed the STRING database to generate a network of protein–protein interactions, according to previous studies [31]. The cut-off criterion for inclusion in the network was that the median confidence level of the interaction score was 0.400. Figure 4A shows all 85 nodes and 50 edges in the protein–protein-interaction network, where the average node degree was 1.18. The Cytohubba plug-in of Cytoscape was used to select the potential hub genes. The following 10 genes had the highest net degree ranking: *LOC689899, Fau, Rpl7a, LOC100360491, Rps19, LOC685085, AC136661.1, Fos, Ebna1bp2, Egr1* (Figure 4B and Table 3).

**TABLE 3:** The hub genes selected by cytohubba.

Hub genes	log2FoldChange	False-discovery rate	*p*-value
*LOC689899*	6.136147593	1	1.03E-16
*Rpl7a*	0.606298027	1	0.003766165
*LOC100360491*	1.764732106	0.146915516	0.000136002
*Rps19*	−1.166814696	1	0.004378735
*LOC685085*	−1.306683174	1	0.032988997
*Fau*	1.848475885	0.061686258	5.05E-05
*AC136661.1*	1.783174086	0.37144022	0.025586079
*Ebna1bp2*	−0.817818978	0.967933878	0.002607751
*Egr1*	−0.64586611	0.056699571	3.26E-05
*Fos*	−1.006387096	0.004093582	1.83E-06

### Verification of the hub genes

**FIGURE 5:**
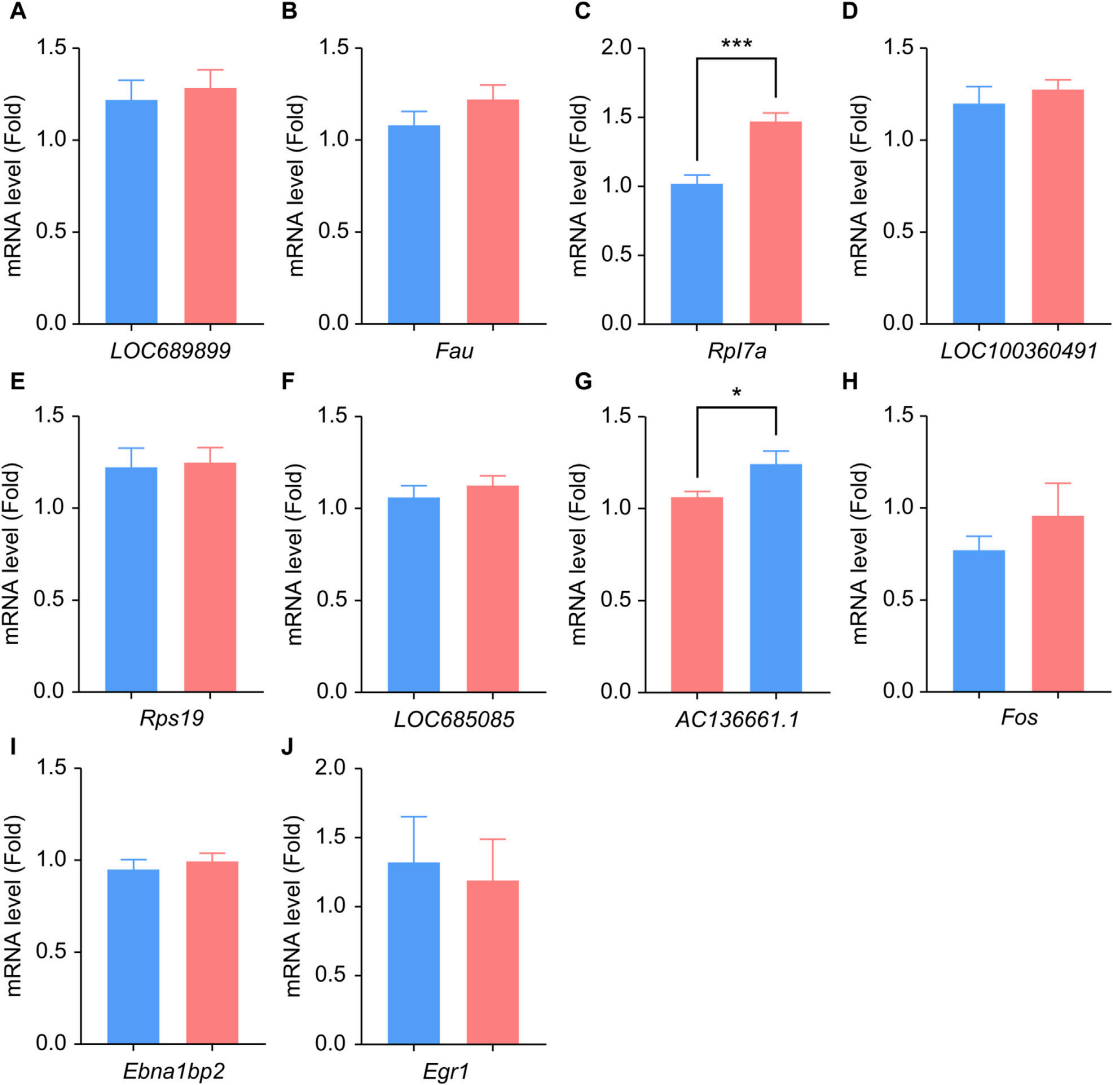
Results of qPCR analysis of the selected hub genes. **(A–J)** Differences in the mRNA-expression levels of ten key genes between the non-tinnitus and tinnitus groups are shown, including *LOC689899* **(A)**, *Fau* **(B)**, *Rpl7a* **(C)**, *LOC100360491* **(D)**, *Rps19* **(E)**, *LOC685085* **(F)**, *AC136661.1* **(G)**, *Fos* **(H)**, *Ebna1bp2* **(I)** and *Egr1* **(J)**. *n* = 5 animals, *p < 0.05.

We performed qPCR to verify the expression levels of *LOC689899, Fau, Rpl7a, LOC100360491, Rps19, LOC685085, AC136661.1, Fos, Ebna1bp2, and Egr1*. Although LOC689899 had the highest betweenness centrality value, it did not show a significant difference in expression between the two groups (*p* = 0.69, Figure 5A). *Fau* (*p* = 0.23, Figure 5B), *LOC100360491* (*p* = 0.50, Figure 5D), *Rps19* (*p* = 0.80, Figure 5E), *LOC685085* (*p* = 0.43, Figure 5F), *Fos* (*p* = 0.36, Figure 5H), *Ebna1bp2* (*p* = 0.53, Figure 5I) and *Egr1* (*p* = 0.78, Figure 5J) also showed no significant differences. However, *Rpl7a* was expressed at significantly higher levels in the tinnitus group than in the non-tinnitus group (*p* < 0.001, Figure 5C) which was also true for the *AC136661.1* (*p* < 0.05, Figure 5G).

## Discussion

Tinnitus is an intractable condition that impairs quality of life and imposes a heavy burden on society when it progresses to a chronic state. Research to identify the best method to treat tinnitus is still ongoing because of its elusive pathogenic mechanisms. In this study, we first established a rat model of tinnitus by exposing the animals to loud noise, which was subsequently validated by applying gap-induced, PPI of acoustic startle response (GPIAS) methods. Subsequently, genetic changes in the MGB between the non-tinnitus and tinnitus groups were analyzed using RNA-seq. We identified 87 differentially expressed genes (DEGs; 40 upregulated and 47 downregulated genes) that were highly related to tinnitus. GO analysis of the DEGs was performed to identify their potential functions and KEGG analysis was performed to clarify the signaling pathways involved. Ten hub genes were finally identified based on the protein–protein-interaction network analysis and were tested further at the transcriptional level.

In this study, animals showed tinnitus-related behavior after tonal stimulation at 6, 12, and 16 kHz, which was close to the frequencies of the loud noises used in this study. These results were consistent with those of previous research, which showed that the tinnitus frequency was similar to the noise frequency [32]. The most upregulated genes *AABR07046778.1, Ngfr, Nudt10, LOC100360647, AC136661.1, Fau, Sdc1, LOC100360491,LOC100910678, and Snrpg.* The glutamate excitotoxicity associated with mitochondrial dysfunction can cause tinnitus [33]. *AABR07046778.1* may modulate tinnitus by participating the same process. The most downregulated genes in the tinnitus (when compared to the non-tinnitus group) were *LOC103690064, AABR07048397.1, Trpv1, AABR07061378.1, Ncaph, Spata20, Rps19, Enpp3, Grtp1, and Glra1*. *LOC103690064* is functionally similar to m7GpppN-mRNA hydrolase, which control the degradation, decay and turnover of mRNA by removing the 5′ cap structure from the mRNA [34–36]. The finding that *LOC103690064* was downregulated the pathological retention of RNAs or the protective compensation of RNAs in the MGB [37].

One of the most enriched pathway terms identified in this study was coronavirus disease COVID-19 pathway. This pathway mainly correlates with the immunologic derangement as well as the dysfunction of blood-brain barrier (BBB) induced by coronavirus in the central nervous system [38, 39]. Previous results have shown that patients with COVID-19 also display increased severity and incidence rate of tinnitus [40, 41]. It is reasonable to speculate that in tinnitus, increased pathological immune proteins or BBB permeability occur in the MGB. The coronavirus disease COVID-19 is also commonly enriched in neurodegenerative diseases, which is often associated with tinnitus, a tentative sign of neurodegeneration [42]. KEGG pathway terms associated with neuroactive ligand-receptor interaction were highly enriched, which further verifies that tinnitus is one type of neurodegenerative disorder that is highly correlated with the damage of synaptic transmission [43]. Genes involved in neuroactive ligand–receptor interactions, such as *Npy2r, Htr2c, and Rxfp1*, were significantly dysregulated during cognitive dysfunction or memory impairment which was tightly related with tinnitus [44, 45].

Identifying the genes encoding key proteins responsible for noise-induced tinnitus could help elucidate the mechanism responsible for the condition. *Rpl7a* which encodes the ribosomal protein large subunit was found to be upregulated in the tinnitus group compared with the non-tinnitus group. Previous research showed that the levels and activity of ribosomal proteins can change during neurodegeneration as well as brain aging [46–48]. *Rpl7a* were found to promote the growth and regeneration of neural axons after optic nerve crush injury suggesting that the elevated level of *Rpl7a* can facilitate the communication between adjacent neurons and increase the output of MGB to auditory cortex [49]. *AC136661.1* was another up-regulated hub gene in tinnitus group, selected by the protein–protein-interaction network. This gene codes the 40S ribosomal protein S2 (RPS2) in rats, a protein responsible for aminoacyl-tRNA binding. This protein is similar to yeast S4 and *Escherichia coli* S5 ribosomal proteins [50]. Upregulation of *AC136661.1* may permit the fidelity of the translation of mRNA ribosomes in mitochondria which guarantee the supply of energy in MGB neurons [51]. This is a reasonable finding because the main cell subtypes (accounting for >99% of cells in the MGB in rodents) are glutamatergic [52]. The increased activity of excitatory neurons led to an increased output from the MGB to the auditory cortex in a model of central gain, which showed increased excitability of neurons in the auditory cortex [53, 54].

Previous research revealed the presence of genes related with axonal branching (*ANK2, AKAP9, and TSC2*) in tinnitus patients [20]. These also support our hypothesis that enhanced excitability of neurons occurred in tinnitus as increased branches promote connections among neurons and even brain regions. Other genes, such as *COL11A1, GRK6, MFHAS1, MSRA, XKR6, C8orf12, AF131215.5, and BLK,* which were reported to be correlated with tinnitus-related disorders were not observed in our study [55]. This may be due to the fact that our study mainly focused on acute tinnitus that is scarcely associated with psychological problems. Meanwhile, other studies have shown that genes such as CACNA1E that influence the firing of neurons are also associated with tinnitus, which is applicable to our study, suggesting the altered excitability of neurons in the brain [56]. Xie *et a*l. identified that genes related with the formation of cilia and infiltration of inflammatory cells also participate in the development of tinnitus [57]. Our study did not find such genetic changes as we only focused on the central nervous system instead of the peripheral nervous system. Further investigations need to be done to clarify the regional effect on the candidate genes related to tinnitus. We did not find the similar genes reported in the human studies by Amanat et al. [20]. However, we found gene (NGFR) shares similar functions with ANK2 which is high imperial for the growth and extension of neurons [58]. In addition, we also did not detect the genes reported in the work by Xie et al such as WNT11 and TNFRSF1A [57]. However, we cannot exclude the possibility that genes in different signaling pathway may interact with each other which indirectly influence the tinnitus. For example, WNT family member could activate NGFR transcription in a ZEB1-dependent manner [59]. Additional work also should be done to verify the hypothesis.

This study has certain limitations that should be acknowledged. First, we only utilized male rats as the target to reduce the effects of sex on hearing levels; however, female rats displayed comparable variability in hearing levels to male rats during the reproductive cycle [60]. Additional studies should be performed with female rats before the results are translated to a clinical study. Second, we predominantly focused on the occurrence (rather than the sustainment) of tinnitus (i.e., the chronic phenotype) [61]. Tinnitus lasted longer in our model (established by exposing rats to loud noises) than drug-induced tinnitus lasts. When considering simulating patients disturbed by tinnitus, these results should be cautiously interpreted because in clinical practice, numerous patients experience tinnitus without explicit pathogenesis but with normal bilateral hearing. Third, although similar genetic variability occurs between rodents and humans, the hub genes selected in our study should be translated cautiously into humans because some human diseases do not occur naturally in rodents [62]. Fourth, the genes encoding proteins related to tinnitus were only confirmed at the transcriptional level. Further studies are required to determine whether these proteins can be translated clinically into effective targets for treating tinnitus.

I In conclusion, we identified 88 DEGs in tinnitus (43 upregulated genes and 45 downregulated genes). Most DEGs enriched were associated with COVID-19 and neuroactive ligand-receptor interaction-related pathways. We selected the genes (*Rpl7a and AC136661.1*), which are highly related to the normal function of ribosome, for further analysis. These findings will contribute significantly to the development of an effective therapeutic approach for tinnitus, resulting in an exciting breakthrough for this debilitating disease.

## Data Availability

The original contributions presented in the study are included in the article/Supplementary material, further inquiries can be directed to the corresponding author/s.

## Footnotes

1
http://stringdb.org/
