# Supplementary figures and images for: Transcriptional-profile changes in the medial geniculate body after noise-induced tinnitus
Source: Exp Biol Med (Maywood). 2024 Mar 18;249:10057. doi: 10.3389/ebm.2024.10057 (PMC10984379; doi:10.3389/ebm.2024.10057)

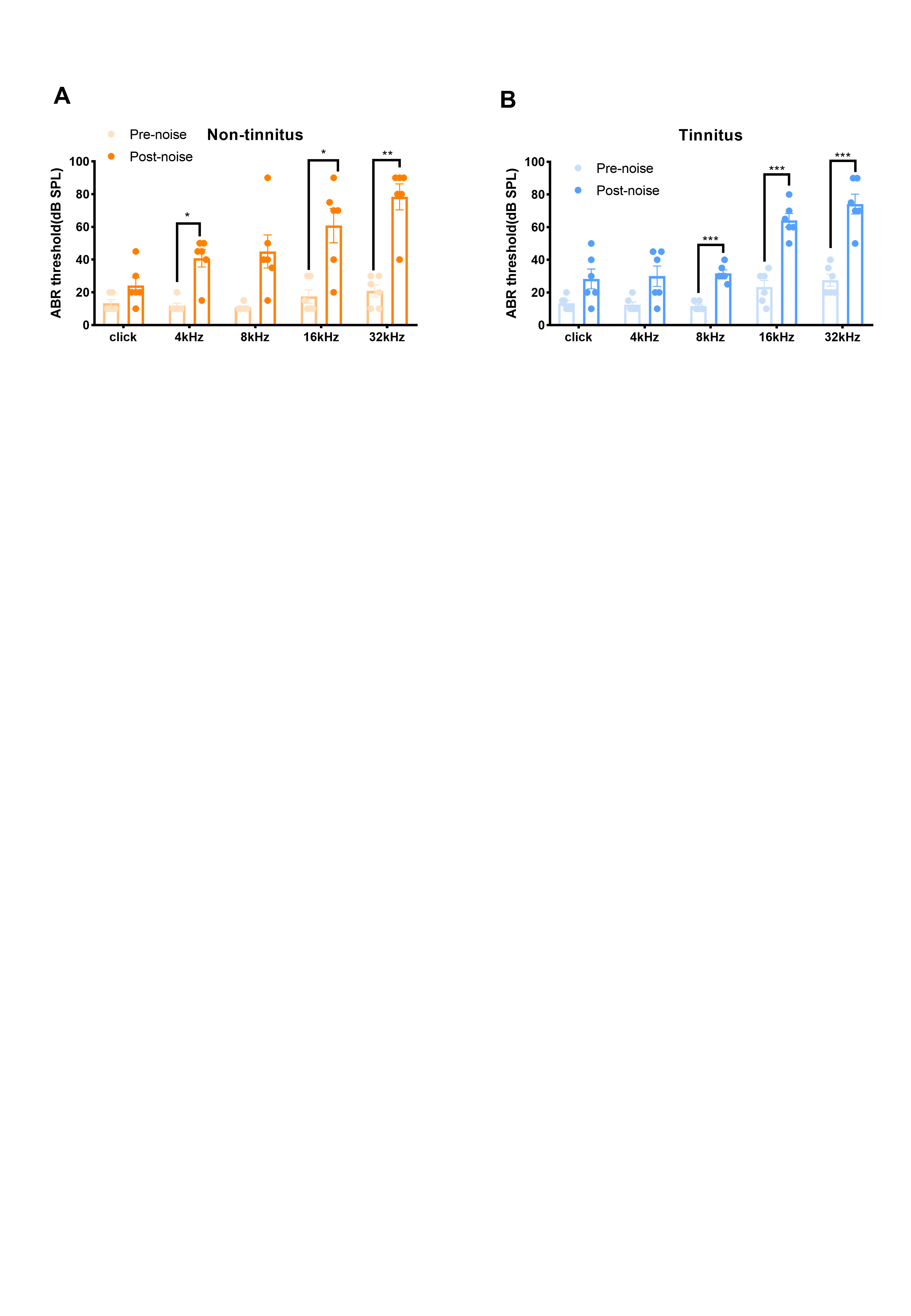

Supplement: Supplementary file 1 [file Image1.TIF]
